# Interaction Between Dietary Fiber Intake and *MTNR1B* rs10830963 Polymorphism on Glycemic Profiles in Young Brazilian Adults

**DOI:** 10.3390/genes16050497

**Published:** 2025-04-27

**Authors:** Ana Carolina da Silva Lima, Nathália Teixeira Cruvinel, Nara Rubia da Silva, Marcela Moraes Mendes, Amélia Cristina Stival Duarte, Alexandre Siqueira Guedes Coelho, Karani S. Vimaleswaran, Maria Aderuza Horst

**Affiliations:** 1 Nutritional Genomics Research Group, Faculty of Nutrition, Federal University of Goiás, Goiania 74605-080, GO, Brazil; anacarolinalima@ufg.br (A.C.d.S.L.); nathycruvinel08@gmail.com (N.T.C.); nutrinara@discente.ufg.br (N.R.d.S.); mendesmarcelam@ufg.br (M.M.M.); amelia_duarte@egresso.ufg.br (A.C.S.D.); 2 Health Research Coordination, Organization: State Department of Health from Goiás (SES-GO), Goiânia 74853-070, GO, Brazil; 3 School of Agronomy, Federal University of Goias, Goiânia 74690-900, GO, Brazil; alexandre_coelho@ufg.br; 4 Hugh Sinclair Unit of Human Nutrition, Department of Food and Nutritional Sciences, University of Reading, Reading RG6 6DZ, UK; 5 The Institute for Food, Nutrition, and Health, University of Reading, Reading RG6 6AH, UK

**Keywords:** food intake, glycemia, fiber, nutrigenetics, precision nutrition

## Abstract

Background/Objective: The single-nucleotide polymorphism (SNP) rs10830963 in the melatonin receptor 1B (*MTNR1B*) gene influences insulin secretion and glucose metabolism and has been associated with an increased risk of type-2 diabetes. This study aimed to explore the interaction between dietary intake and the *MTNR1B* rs10830963 polymorphism on glycemic profiles in young Brazilian adults. Methods: This cross-sectional study assessed 200 healthy young adults (19–24 years), evaluating the *MTNR1B* rs10830963 genotype, anthropometric parameters, glycemic markers (fasting insulin, glucose, HOMA-IR, and HOMA-β), and dietary intake via three 24 h dietary recalls. Genotype–diet interactions were tested using multivariate linear regression models adjusted for confounders. Results: The carriers of the G allele exhibited a positive association with fasting insulin levels (*p* = 0.003), insulin/glucose ratio (*p* = 0.004), HOMA-IR (*p* = 0.003), and HOMA-β (*p* = 0.018). Energy-adjusted fiber intake showed a significant genotype-specific interaction only in carriers of the G allele, where higher dietary fiber intake was significantly associated with lower fasting insulin (*p_interaction_* = 0.034) and HOMA-IR (*p_interaction_* = 0.028). Conclusion: Our findings indicate that the *MTNR1B* rs10830963 polymorphism is associated with glycemic markers, and dietary fiber intake may attenuate the adverse effects of the *MTNR1B* rs10830963 G allele on glycemic profiles in young Brazilian adults. This highlights the potential role of fiber in improving health outcomes for individuals carrying this risk allele. To validate these results and assess the broader implications for the Brazilian population, further intervention studies and larger-scale research are essential.

## 1. Introduction

Over the past few decades, there has been a global increase in metabolic disorders, such as obesity and type 2 diabetes mellitus (T2DM) [1,2,3]. Specifically, the prevalence of T2DM has been escalating worldwide in recent times. According to the International Diabetes Federation (IDF), there is a projected rise in the global diabetic population from 537 million in 2021 to 783 million by 2045 [3]. Brazil is the sixth country with the highest number of individuals diagnosed with diabetes, reaching 15.7 million [3]. Multiple factors contribute to the rising incidence of metabolic disorders, including poor dietary choices, genetic predisposition, and physical inactivity [4,5]. Consuming an excessive high-calorie and low-fiber diet can result in insulin resistance and weight gain, both of which are linked to the development of T2DM [6,7]. In addition, genetic factors play a crucial role in interacting with diet, as studies have indicated that single nucleotide polymorphisms (SNPs) in metabolism-related genes can elevate the risk of T2DM [8,9,10].

Specifically, studies have investigated the association between the rs10830963 polymorphism of the melatonin receptor 1 B (*MTNR1B*) gene and various metabolic and glycemic traits [11,12,13,14]. The investigation conducted by the Trans-Omics for Precision Medicine (TOPMed) program in over 23,000 non-diabetic individuals from diverse races/ethnicities/populations revealed a strong association between rs10830963 and T2DM [15]. Li et al. [16] conducted a meta-analysis involving 13,752 participants and discovered that individuals carrying the G allele in the *MTNR1B* rs10830963 had higher levels of fasting glycemia and insulin resistance, making them more susceptible to T2DM. Emerging evidence indicates that dietary factors may modulate the metabolic effects of the *MTNR1B* rs10830963 polymorphism. Diets high in carbohydrates and glycemic index were shown to be associated with exacerbated glycemic responses, particularly among carriers of the G risk allele [17]. Moreover, the Brazilian population exhibits distinct dietary patterns that may influence gene–diet interactions. National dietary surveys have reported a high intake of processed and ultra-processed foods, alongside inadequate consumption of dietary fiber, particularly among adults [18]. These patterns coexist with an increasing prevalence of overweight and obesity [19], which may exacerbate genetic susceptibility to metabolic disturbances. Such contextual factors highlight the importance of investigating gene–diet interactions within this population to inform targeted nutritional strategies.

To date, in the Brazilian population, there is only one study on *MTNR1B* SNP rs10830963, where women carrying the G allele were 1.4 times more likely to develop gestational diabetes than those carrying the C allele [20]. However, this study did not assess gene–diet interactions. Understanding the connections between *MTNR1B* SNP, dietary intake, and glycemic profiles in young Brazilian adults can prove beneficial for developing precise nutritional approaches aimed at preventing or managing adult-onset metabolic disorders. Therefore, this study aimed to examine the association of *MTNR1B* rs10830963 with glycemic profiles and the interaction of the SNP with dietary intake in young Brazilian adults.

## 2. Methods

### 2.1. Participants and Anthropometric and Sociodemographic Measurements

This is a cross-sectional study that analyzed data from two hundred healthy young adults aged between 19 and 24 years, with no diagnosis of non-communicable chronic diseases, recruited for the “Obesity, Lifestyle, and Diabetes (BOLD)” study at the Federal University of Goiás (UFG) between March and June 2019 [21,22,23]. Participants were recruited through print and digital advertisements placed on college campuses and on social media. Upon enrollment, all participants completed a baseline questionnaire capturing information on health status, socioeconomic factors, and demographics. Exclusion criteria included individuals who were undergoing nutritional monitoring, taking hypoglycemic drugs, or using vitamins and mineral supplements. Those undergoing menopause or hormone replacement therapy; those with acute clinical conditions such as infection, inflammation, fever, or diarrhea; or those with self-reported autoimmune or chronic diseases, including type 1 and T2DM, moderate/severe hypertension, cancer, or rheumatoid arthritis were excluded from this study. Further details of the BOLD study have been published previously [21,22,23]. This study was conducted according to the guidelines established in the Declaration of Helsinki [24], and ethical approval was granted by the Research Ethics Committee of the Federal University of Goiás (approval number: 3.007.45608/11/2018). Each participant provided informed consent prior to participation.

The anthropometric, sociodemographic, and lifestyle questionnaires were performed by a trained researcher according to details published elsewhere [21,22,23]. Body composition was performed by Dual Energy Radiological Absorptiometry (DXA) using the Lunar model DPX NT (General Electric Medical Systems Lunar^®^; Madison, WI, USA).

### 2.2. Biochemical Measurements

Peripheral venipuncture was used to collect 5 mL of blood from the participants in the morning, following a 12 h fast and a 72 h abstention from alcohol consumption. Participants’ blood was collected using suitable tubes containing the anticoagulant ethylenediaminetetraacetic acid (EDTA) and was promptly processed at the Rômulo Rocha Laboratory (Goiânia, Brazil). Plasma insulin and glucose levels were determined by the enzymatic colorimetric technique, with an automatic System Vitros Chemistry 950 XRL (Johnson & Johnson, New Brunswick, NJ, USA). Plasma glycated hemoglobin (HbA1c) was measured using high-pressure liquid chromatography (HPLC-Bio-Rad Laboratories, Hercules, NY, USA). The Homeostasis Model Assessment Estimate of Insulin Resistance (HOMA-IR) and β-cell Function (HOMA-β) were calculated using the HOMA2 Calculator (©Diabetes Trials Unit, University of Oxford, Oxford, UK, version 2.2.3).

### 2.3. Dietary Intake

Dietary intake was assessed by a trained nutritionist using three 24 h food records consisting of non-consecutive days, including one weekend [25]. The multiple-pass method, developed by the United States Department of Agriculture (USDA), was employed to improve recall accuracy and reduce recall bias. This structured approach consists of five steps to collect dietary data: (1) an uninterrupted listing of all foods and beverages consumed; (2) an investigation into foods that are commonly forgotten; (3) questions about the time and occasion of each item; (4) detailed descriptions, including amounts and method of preparation of the food; and (5) a final review to check the accuracy of the responses [26]. The nutritionist conducted the first interview in person and the following two interviews were conducted via phone calls. All reported food quantities were converted to grams using the Avanutri Online^®^ diet calculation software (Avanutri Informática Ltda., Rio de Janeiro, Brazil).

### 2.4. Genotyping

The *MTNR1B* rs10830963 SNP, known for its association with glycemic profile [13,14,15,16,17], was selected for genotyping. Blood samples (3 mL) were collected in ethylenediaminetetraacetic acid tubes (BD Vacutainer ^®^ tubes, Franklin Lakes, USA) and transported in a temperature-controlled environment (−80 °C) by the World Courier Company from Brazil to LGC Genomics, London, UK, for genotyping. Genomic DNA was extracted from peripheral blood leukocytes using standard salting-out. Genotyping of the *MTNR1B* rs10830963 SNP was performed using the competitive allele-specific PCR-KASP^®^ assay, a fluorescence-based genotyping technology that employs allele-specific primers and a common reverse primer to detect SNP variants with high accuracy [27].

### 2.5. Statistical Analysis

The database underwent double-entry typing, and the data were validated. Data distribution was evaluated using the Shapiro–Wilk test. Continuous variables were presented as means and standard deviations. The Mann–Whitney U test was used to compare the data. Categorical data were presented as relative frequencies (%). Hardy–Weinberg equilibrium (HWE) was assessed using the chi-square test. Nutrient intake was energy-adjusted using the residual method [28] with the purpose of estimating dietary intake regardless of the energy intake. Multiple linear regression with backward strategy and adjustment for confounding variables (age, sex, body mass index, and level of physical activity) were used to investigate the associations between the *MTNR1B* rs10830963 genotypes and the glycemic profile variables, such as insulin, HOMA-IR, HOMA-β, the insulin/glucose ratio, and glycated hemoglobin. Interactions of the SNP with dietary factors on glycemic profiles were also tested. The data were processed using R software (version 4.1.3). A level of 5% was considered statistically significant (*p* < 0.05).

The power of the test was calculated based on an effect size of 0.3567 derived from the R^2^ for the interaction (SNP rs10830963 × dietary fiber on fasting insulin) in our linear regression model adjusted for confounding variables, for a sample of 200 individuals, and a significance level of 0.05. The power obtained was equivalent to 99%. The software used was G-Power^®^ (version 3.1.9.6).

## 3. Results

The *MTNR1B* SNP rs10830963 was in the Hardy–Weinberg equilibrium (*p* = 0.268), and the minor allele frequency (MAF) was 0.18. All participants were of Brazilian ancestry. Participants were mainly women (73.5%), with a mean age of 21.3 ± 1.7 years, and a mean BMI of 25.2 ± 4.7 kg/m^2^ for the total sample. Participants’ characteristics (anthropometric and dietary intake) were compared between carriers of the CC genotype and G allele carriers (CG and GG genotypes), as summarized in Table 1.

Table 2 shows the association of *MTNR1B* SNP rs10830963 with glycemic profile and anthropometric measurements. The *MTNR1B* SNP rs10830963 was positively associated with fasting insulin (*p* = 0.003), the insulin/glucose ratio (*p* = 0.004), HOMA-IR (*p* = 0.003), and HOMA-β (*p* = 0.018). No significant association (*p >* 0.05) was observed with glycated hemoglobin or fasting glucose.

Table 3 presents the interactions between *MTNR1B* rs10830963 genotypes and dietary intake on glycemic profile. There was significant interaction between the *MTNR1B* rs10830963 genotypes and fiber consumption on the glycemic profile markers fasting insulin (*p_interaction_* = 0.011), the insulin/glucose ratio (*p_interaction_* = 0.016), and HOMA-IR (*p_interaction_* = 0.010). No significant interactions (*p_interaction_ >* 0.05) were observed for the remaining variables.

**Figure 1:**
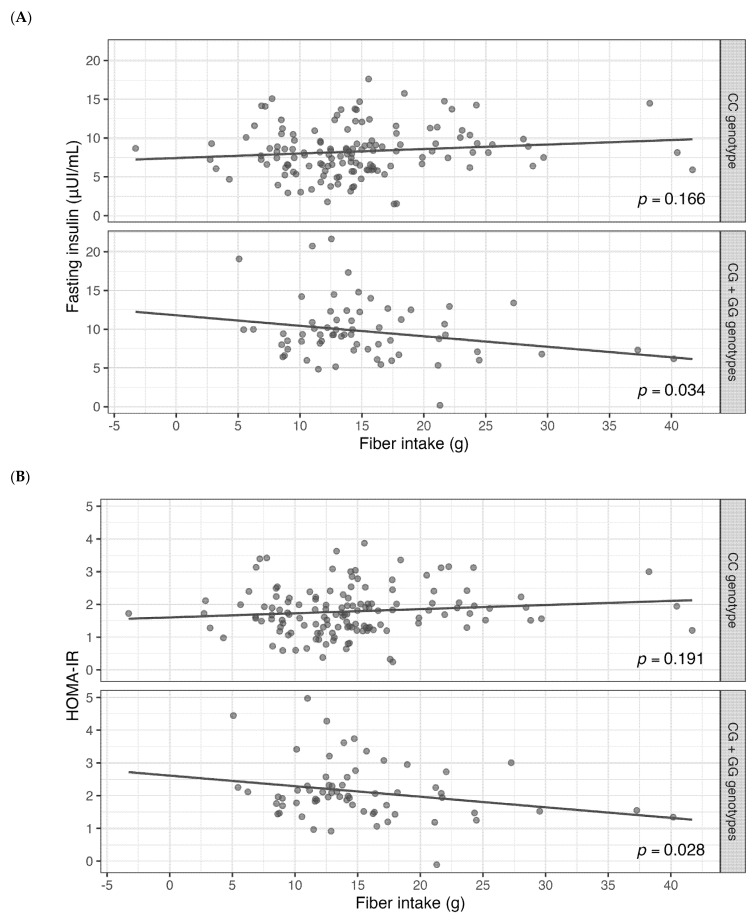

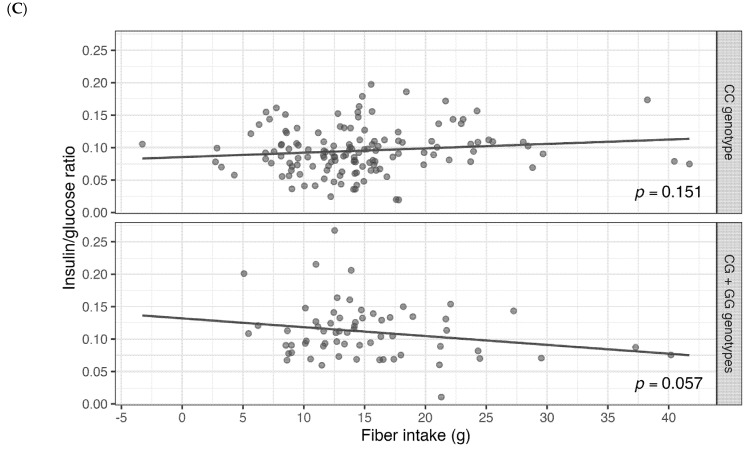
Interaction between the *MTNR1B* rs10830963 genotype and dietary fiber intake on glycemic markers. (**A**) Interaction effect on fasting insulin; (**B**) interaction effect on the HOMA-IR index; (**C**) interaction effect on the fasting insulin/glucose ratio. Individuals are stratified by genotype: CC genotype (no risk allele) vs. CG + GG genotypes (at least one risk allele). Fiber intake (x-axis) is presented as residuals after adjusting for total energy intake, resulting in negative values when intake is below the group mean. Glycemic outcomes (y-axis) are adjusted for mean age, body mass index (BMI), and carbohydrate intake. Regression lines indicate adjusted relationships within each genotype subgroup. HOMA-IR, homeostasis model assessment of insulin resistance.

To clarify the interaction direction, we conducted a genotype-stratified analysis, as illustrated in Figure 1A–C. This analysis evaluated the relationship between dietary fiber intake and glycemic markers, adjusted for mean age, BMI, and carbohydrate intake. The fiber consumption values on the x-axis are presented as residuals adjusted for mean caloric intake. Individuals carrying at least one G risk allele (CG or GG genotype) of *MTNR1B* rs10830963 exhibited a significant negative association between calorie-adjusted fiber intake and fasting insulin (*p* = 0.034), as well as HOMA-IR (*p* = 0.028), and a trend for the insulin/glucose ratio (*p* = 0.057). These results suggest that higher fiber intake, in relation to overall caloric intake, is linked to better insulin sensitivity among carriers of the G allele. In contrast, individuals with no risk allele (CC genotype) showed no significant associations.

## 4. Discussion

To the best of our knowledge, this is the first study to demonstrate that in young Brazilian adults, fiber consumption may modify the association between the *MTNR1B* rs10830963 and glycemic profile. Individuals carrying the G allele (CG + GG genotypes) exhibited less favorable insulin-related parameters compared to CC homozygotes, supporting the notion that the G allele acts as a risk allele for T2DM, even among younger individuals, which is consistent with the existing literature in other populations [11,12,13,14,29]. Additionally, among G allele carriers, higher fiber intake was inversely associated with fasting insulin levels and HOMA-IR; in contrast, this relationship was not observed in individuals with the CC genotype. Considering that most Brazilians consume substantially less fiber than the recommended 25–38 g/day [18,30], our findings underscore the potential impact of adequate fiber intake in attenuating insulin resistance, particularly among those with a genetic predisposition to T2DM.

Remarkably, despite being young, 29.5% of participants had at least one altered glycemic profile marker, emphasizing the relevance of early identification and targeted nutritional interventions during young adulthood, a critical period for establishing lifelong dietary habits and preventing chronic diseases. Supporting the clinical relevance of our findings, a meta-analysis involving over 170,000 individuals validated that rs10830963 exhibits the strongest association with the risk of T2DM [31]. This meta-analysis included populations of diverse ethnic backgrounds, predominantly of Caucasian descent, followed by East Asian and South Asian groups. The results demonstrated a significant association between the G allele and increased risk of T2DM in the overall analysis, with the strongest effect observed among Caucasians, while no significant associations were detected in the East Asian or South Asian populations [31]. Additionally, the influence on fasting plasma glucose levels was also confirmed in children and adolescents, indicating an early effect of the rs10830963 G risk allele during development [32,33]. Our findings support the current evidence by demonstrating that genetic variation in the *MTNR1B* rs10830963 is significantly associated with early risk markers of metabolic dysregulation related to T2DM, even among young and healthy adults. Consistent with previous research, *MTNR1B* rs10830963 has been linked to impaired insulin secretion and altered glucose homeostasis, which can predispose individuals carrying the G allele to increased insulin resistance risk [34,35]. Specifically, studies have reported that the G allele is associated with reduced β-cell function and increased fasting glucose levels, highlighting a potential genetic vulnerability in glucose metabolism [12,32,36].

A key finding of our study is the significant interaction between the *MTNR1B* rs10830963 and dietary fiber intake on glycemic profile markers. Few SNPs have been evaluated in gene–diet interaction studies regarding fiber and risk factors for T2DM. The most relevant finding in the literature is that polymorphisms in the *TCF7L2* gene have been shown to be associated with glucose metabolism markers when dietary fiber consumption is increased. This suggests an underlying mechanism whereby higher fiber intake promotes favorable glucose homeostasis [37]. Our findings build upon this evidence by demonstrating that Brazilian carriers of the *MTNR1B* rs10830963 G allele may obtain specific metabolic benefits from fiber-rich diets.

The *MTNR1B* gene encodes melatonin receptor type 2 (MT2), which is a receptor integral to the circadian regulation of insulin secretion and glucose homeostasis and one of two high-affinity receptors for melatonin [38,39]. Melatonin acts primarily by binding to its receptors (MT1 and MT2), which are expressed in pancreatic β-cells, influencing insulin secretion rhythms and overall metabolic control. The rs10830963 variant is common and located within the single 11 kb intron of the *MTNR1B* gene, and the SNP has been associated with altered receptor expression or signaling efficiency, potentially leading to impaired β-cell function, reduced insulin secretion, and consequent insulin resistance [38,39,40]. A study with 26,576 individuals from Candidate-gene Association Resource (CARe), assessed the relationship of the *MTNR1B* rs10830963 G allele with the likelihood of healthy individuals developing glucose intolerance (prediabetes) and of those with intolerance progressing to diabetes. Interestingly, the G risk allele was more strongly associated with the transition from normoglycemia to prediabetes than with the progression from prediabetes to diabetes [41]. Further in-depth research on insulin secretion revealed a connection between the rs10830963-G allele and reduced early-phase insulin secretion, mirroring the impairment seen in patients with type 2 diabetes [34]. This mechanistic pathway aligns closely with our findings, where young adult carriers of the G allele presented higher fasting insulin levels and increased insulin resistance markers compared to CC homozygotes. Furthermore, we observed a significant gene–diet interaction, where individuals carrying the G allele who consumed greater amounts of dietary fiber showed more favorable insulin sensitivity markers. Dietary fiber intake is known to attenuate postprandial glycemic excursions, enhance insulin sensitivity, and promote a beneficial gut microbiota composition, all of which may contribute to reducing susceptibility to metabolic disturbances [42].

Beyond promising insights for precision nutrition, our findings also present important implications for public health strategies in Brazil. Current fiber consumption among Brazilian adults (~15 g/day on average) remains substantially below the recommended intake of 25–38 g/day, reflecting a widespread dietary gap that may contribute to increased metabolic risk [18,30]. Our results specifically highlight that individuals carrying the *MTNR1B* rs10830963 G allele who consume more fiber have better glycemic profiles. This reinforces the public health message that adopting a fiber-rich diet may be an actionable and cost-effective nutritional modification to attenuate genetic susceptibility to insulin resistance.

Young adulthood represents a critical window of opportunity for detecting early metabolic dysregulation before overt clinical manifestations arise [43]. In a recent study on the ancestral genomic architecture of glycemic traits, various SNPs were associated with fasting glucose, 2 h glucose following an oral glucose challenge, HbA1c, and fasting insulin. Notably, the *MTNR1B* rs10830963 SNP was linked to both HbA1c and fasting glucose; however, none of the markers showed an association with this SNP in our study. The most likely explanation for this discrepancy is the age of the individuals assessed, which varied among the studies [44], and most of them had a mean age above 40 years. Unlike older populations, who may already exhibit established insulin resistance or T2DM, younger adults (from 19 to 24 years old) are typically asymptomatic, making subtle metabolic disturbances easier to overlook in routine clinical assessments [43,45,46]. In this context, Sorlí et al. [29] demonstrated that the presence of the G allele in the *MTNR1B* rs10830963 polymorphism has a significantly stronger impact on fasting glucose levels in younger individuals compared to older adults, highlighting the greater genetic susceptibility in this age group and emphasizing the critical importance of early identification of the genetic risk factor. By identifying high-risk genotypes and modifiable dietary factors—such as fiber intake—during early adulthood, preventive interventions can be proactively implemented, potentially slowing or halting disease progression.

Our study has some limitations that should be acknowledged. First, its cross-sectional design restricts our ability to infer causal relationships among genetic variation, dietary factors, and metabolic outcomes. To partially overcome this, we applied robust statistical methodologies, such as multivariate regression adjusting for critical confounders, and strictly adhered to standardized data collection protocols to minimize the risk of spurious associations. Second, our relatively small sample size may affect the generalizability of our findings. Nevertheless, we selected a well-characterized cohort of healthy young adults with clearly defined inclusion criteria, thus enhancing internal validity and reducing potential bias arising from heterogeneous health statuses. Third, unmeasured environmental influences, such as circadian disruption or sleep quality, may impact melatonin signaling and glycemic control. Future studies should consider incorporating more comprehensive evaluations of sleep patterns and circadian rhythms, to further delineate these environmental influences. Despite these limitations, our study holds strengths, including rigorous dietary assessment through multiple 24 h recalls, comprehensive training of the research team, and a focused investigation of young adults—a population in which subclinical metabolic alterations frequently go unnoticed. By identifying genetic risks at an early stage in young individuals, our results provide a robust foundation for longitudinal research, paving the way for preventive strategies which are capable of delaying or averting the progression toward overt metabolic diseases.

## 5. Conclusions

In conclusion, our study provides evidence that the *MTNR1B* rs10830963 polymorphism is significantly associated with early markers of insulin resistance in young Brazilian adults. Furthermore, we identified a notable gene–diet interaction, indicating that individuals carrying at least one copy of the G allele may derive specific metabolic benefits from higher dietary fiber consumption. These findings highlight the potential value of personalized dietary interventions, particularly fiber-rich interventions, to improve insulin sensitivity and mitigate genetic susceptibility. Future studies in larger and ethnically diverse populations, incorporating comprehensive assessments of both genetic and lifestyle factors, will be essential to confirm these associations and refine precision nutrition strategies. Ultimately, integrating genetic information into targeted nutritional recommendations offers a promising pathway for the prevention and early management of metabolic diseases, including T2DM, especially among genetically vulnerable young adults.

## Figures and Tables

**Table 1:** Anthropometric and dietary characteristics of healthy young Brazilian adults stratified by the *MTNR1B* rs10830963 genotype.

Characteristics of Study Participants	*MTNR1B* (rs10830963)		*p*
	CC (N = 136)	CG + GG (N = 64)	
Age (years)	21.3 ± 1.6	21.5 ± 1.8	0.504
BMI (kg/m^2^)	23.4 ± 4.5	23.3 ± 4.3	0.910
WC (cm)	74.8 ± 12.4	74.0 ± 15.7	0.738
Body fat mass (%)	33.6 ± 11.2	34.7 ± 9.5	0.771
Lean body mass (%)	63.6 ± 10.6	62.6 ± 9.0	0.787
Total energy intake (kcal)	1836.2 ± 587.0	1809.9 ± 624.0	0.570
Total protein (g)	77.1 ± 28.6	78.2 ± 29.6	0.974
Total carbohydrate (g)	234.8 ± 84.2	235.9 ± 90.2	0.920
Total fat (g)	65.5 ± 23.5	61.5 ± 22.3	0.216
Dietary fiber (g)	14.7 ± 8.3	15.08 ± 9.1	0.848

Values are expressed as mean ± standard deviation (SD). Individuals are stratified by genotype: CC genotype (no risk allele) vs. CG or GG genotypes (at least one risk allele). Comparisons between genotype groups were performed using the Mann–Whitney U test. BMI, body mass index; WC, waist circumference.

**Table 2:** Association between the *MTNR1B* rs10830963 genotype and glycemic profile markers in healthy young Brazilian adults.

	β	*p*
Fasting insulin (μUI/mL)	1.57	**0.003**
Fasting glucose (mg/dL)	0.57	0.573
Insulin/glucose ratio	0.02	**0.004**
HbA1c (%)	−0.01	0.723
HOMA-IR	0.35	**0.003**
HOMA-β	22.82	**0.018**

Results are presented as β and *p*-values obtained from multiple linear regression analyses adjusted for age, sex, body mass index (BMI), and physical activity level. HbA1c, glycated hemoglobin; HOMA-IR, homeostasis model assessment of insulin resistance; HOMA-β, homeostasis model assessment of β-cell function. Significant associations are highlighted in bold.

**Table 3:** Interactions between the *MTNR1B* rs10830963 genotype and dietary intake (energy and macronutrients) on glycemic markers.

	Energy	Protein	Carbohydrate	Fat	Fiber
	*p_interaction_*	*p_interaction_*	*p_interaction_*	*p_interaction_*	*p_interaction_*
Fasting insulin (μUI/mL)	0.717	0.523	0.638	0.888	**0.011**
Fasting glucose (mg/dL)	0.164	0.538	0.584	0.798	0.254
Insulin/glucose ratio	0.376	0.603	0.623	0.828	**0.016**
HbA1c (%)	0.604	0.347	0.369	0.519	0.517
HOMA-IR	0.985	0.525	0.713	0.960	**0.010**
HOMA-β	0.059	0.720	0.658	0.776	0.093

Values represent *p_interaction_* obtained from multiple linear regression analyses adjusted for age, sex, body mass index (BMI), physical activity level, and carbohydrate intake (specifically for fiber analysis). HbA1c, glycated hemoglobin; HOMA-IR, homeostasis model assessment of insulin resistance; HOMA-β, homeostasis model assessment of β-cell function. Significant interactions (*p* < 0.05) are highlighted in bold.

## Data Availability

All data and analyses are available upon request.
